# Use of home and community-based services and loneliness in older people with functional limitations: a cross-sectional study

**DOI:** 10.1186/s12888-023-05225-6

**Published:** 2023-10-04

**Authors:** Jinxin Zhang, Xiaojie Sun, Aaron Yao

**Affiliations:** 1 Centre for Health Management and Policy Research, School of Public Health, Cheeloo College of Medicine, Shandong University, 44 Wenhuaxi Rd, Jinan, Shandong 250012 China; 2 NHC Key Lab of Health Economics and Policy Research, Shandong University, Jinan, 250012 China; 3 Home Centered Care Institute, Schaumburg, IL USA; 4 University of Virginia, Charlottesville, VA USA

**Keywords:** Loneliness, Home and community-based services, Activities of daily living, Older adults, China

## Abstract

**Background:**

Loneliness is one of the major health problems among older adults. Among this population, home and community-based services (HCBS) have become increasingly popular. Despite its health benefits, little is known about the relationship between HCBS use and loneliness in older people with functional limitations. We aim to explore the characteristics of loneliness among older people with functional limitations and examine the association between HCBS use and loneliness in China.

**Methods:**

We used a cross-sectional data from the 2018 China Health and Retirement Longitudinal Study, which includes a nationally representative sample of Chinese residents aged 65 and older with functional limitations. Logistic regression models were used to examine the associations between HCBS use and loneliness, and we further used propensity score matching to address potential sample selection bias.

**Result:**

In China, 46% of older people with functional limitations felt lonely and only 22% of older people with functional limitations reported using HCBS in 2018. Compared with participants who did not receive HCBS, those who received HCBS were less likely to report loneliness (OR = 0.81, 95% CI = 0.63, 0.99, *p* = 0.048), and the results remained significant after addressing sample selection bias using propensity score matching.

**Conclusion:**

Our results showed that loneliness was common among Chinese older people with functional limitations, and the proportion of HCBS use was low. There was robust evidence to support that among older people with functional limitations, HCBS use was associated with decreased loneliness. Further policies should promote the development of broader HCBS use for older people with functional limitations to reduce their loneliness.

**Supplementary Information:**

The online version contains supplementary material available at 10.1186/s12888-023-05225-6.

## Introduction

Several countries are currently experiencing significant demographic transitions, characterized by a substantial increase in their aging populations [1]. Among these nations, China stands out notably, as it has the world’s largest population of older individuals [1]. With the growth in the aging population and extended life expectancy, the number of older adults with functional limitations is rising sharply in China. Functional limitations are defined as encountering difficulties in independently performing at least one activity of daily living (ADL) or instrumental activity of daily living (IADL) task [2]. Between 2010 and 2020, the number of older adults with functional limitations in China increased from 33 million to 40 million [3, 4], and projections indicate that this figure will further escalate to 140 million by 2035 [5]. Previous studies show that older adults with functional limitations tend to have reduced interaction with family and friends, along with increased vulnerability within their formal networks [6]. Due to their lack of social support and infrequent social contacts [7], they often experience heightened feelings of loneliness [8].

Loneliness is a prevalent and serious mental health issue that deserves attention [9, 10]. Loneliness is a distressing subjective feeling that reflects dissatisfaction with the frequency and closeness of social contacts [11]. Frequently experiencing loneliness has a direct impact on individuals and can also have broader effects on society. Empirical studies have shown that loneliness is associated with several subsequent adverse health outcomes, such as increased risk of dementia [12], poor behavioral and health outcomes [13], increased risk of mortality [14], and ultimately increased substantial social and economic costs [15, 16]. Therefore, the development of interventions to reduce loneliness in older people with functional limitations holds important implications for clinical practice and public health.

Parallel to the demographic aging, China is concurrently experiencing a reduction in family sizes and alterations in co-residence arrangements, driven by urbanization and industrialization. These dynamics have gradually diminished the family’s ability to support the growing elderly population [17]. These transformations present a significant challenge to the intrinsic responsibility of family members to meet the routine caregiving and spiritual needs of their parents [17]. Consequently, to effectively address the care needs of the majority of older adults in China [18], home and community-based services (HCBS) provide an opportunity to reach patients in home and community settings before they require higher levels of institutional care and incur higher medical expenditures [19, 20].

Home and community-based services (HCBS) are a way of providing long-term services and support to older people living outside of institutions [20–22]. In China, home and community-based services (HCBS) can be provided by various sources, including government agencies, nonprofit organizations, and private entities. The providers of HCBS may include medical professionals, social workers, or other trained personnel, depending on the specific services required. The financial cost of HCBS varies, with options for both government-sponsored coverage and private self-payment. The cost may vary depending on the type and scope of services received. Additionally, disparities arise in the expenses borne by households across various regions due to differences in compensation and reimbursement policies. Concurrently, the Chinese government plays a role in providing and regulating HCBS. Nowadays, HCBS have become increasingly popular among China’s older population, and the Chinese central government has issued many policy documents to promote the development of HCBS [21, 22]. Understanding the characteristics of loneliness among older adults with functional limitations and exploring the relationship between home and community-based services (HCBS) use and loneliness are important targets for policymakers, to inform the adjustment of health policy priorities and care items in a timely manner.

Worldwide, empirical studies have reported that HCBS were particularly beneficial in the management of common physical and psychological symptoms among older adults in general [23–25]. However, very few studies focus on understanding the characteristics of loneliness in older adults with functional limitations, and knowledge gaps still exist in the association between HCBS and loneliness. First, limited by qualitative inductive design, previous studies have focused on describing perspectives of older adults with functional limitations on their experience of loneliness during home-based rehabilitation [26], but we know little about the loneliness of older adults with functional limitations in China. Also, previous studies have reported that those who received HCBS were more likely to have objective social contacts [23–25], without examining the association of HCBS use with specific loneliness. This is important because it is possible to feel lonely even when one is surrounded by people. In addition, there are increasing efforts to explore complex interventions (e.g., psychosocial group courses, media campaigns, information sessions) to reduce loneliness, but only a few have proven to help reduce feelings of loneliness [27]. Few studies have reported alleviating the experience of loneliness through group interventions targeted to specific populations using existing community resources in a real-world context [28, 29].

Considering that previous studies have not specifically focused on loneliness in older adults with functional limitations, and that research regarding the relationship between HCBS and loneliness is limited, the aim of this study was to describe the prevalence of loneliness in older adults with functional limitations, compare their sociodemographic characteristics, and explore the association between receipt of HCBS and loneliness. The findings of our study have the potential to inform future policy implementation concerning the enhancement of home and community-based services (HCBS) within the framework of active and healthy aging.

## Methods

### Data sources and participants

This cross-sectional study used data from the 2018 China Health and Retirement Longitudinal Study (CHARLS). The CHARLS targets Chinese adults aged 45 and older and aims to collect a high-quality set of microdata on households and individuals [30]. It is a prospective cohort survey, with the sample stratified by urban/rural areas and by gross domestic product (GDP). The baseline survey used probability proportional sampling (PPS) with a sample of 12,400 households in 450 communities (villages) from 150 counties in 28 provinces [30]. The CHARLS national baseline survey was conducted in 2011 and has been repeated every two to three years since then. Regarding participants, inclusion criteria were (1) adults with functional limitations who lived at home, and (2) aged 65 years old and above. A total of 2,188 older adults with functional limitations were included in the analysis.

### Measures

#### Functional limitations measures

Older adults’ functional limitations were assessed using the 6-item Activities of Daily Living (ADL) scale (eating, toileting, controlling urination and defecation, dressing, getting in and out of bed, and bathing/showering) [31] and the 5-item Instrumental Activities of Daily Living (IADL) scale (cooking, taking medication, doing housework, shopping, and managing money) [32]. Both the ADL and IADL scales have items with four options: (1) no difficulty in activity, (2) difficulty in activity but can be performed without assistance, (3) difficulty in activity and requires assistance, and (4) completely unable to perform activity without assistance. Following classification, we excluded older people without functional limitations who could perform any activities (ADL and IADL) without assistance [2, 31, 32].

#### Independent variables: home and community-based services

Home and community-based services (HCBS) use was assessed by the question, “Have you ever received the following HCBS?” with the following responses: (1) day care centers, nursing homes, senior meals; (2) regular medical checkups; (3) home visits; (4) family beds (a form of care in which a patient is regularly visited at home by a professional for treatment, and the progress of the service is documented in a medical record); (5) community nursing; (6) health management; (7) entertainment; (8) other services; or (9) none of the above. In our study, the value of this variable was coded as “treatment group” if the respondent selected one or more of the above choices and home care services, otherwise the value of this variable was coded as “control group” if the respondent selected “9) none of the above.” This method has been applied in other existing studies [24, 33, 34].

#### Outcome variables: loneliness

Loneliness was self-reported by older adults using a single item: “Do you feel lonely?” with the following options: (1) rarely or none of the time, (2) some or a little of the time, (3) occasionally or a moderate amount of the time, or (4) most or all of the time. For statistical analyses, this variable was categorized into two groups; if the participant answered “1) rarely or none of the time,” it was coded as “No,” otherwise, it was coded as “Yes.” Although single-item self-reported questions may not be as reliable or valid as multi-item scales, these types of questions are easy to understand and answer. This single-item question about loneliness has been widely used in previous studies [35–37].

#### Confounding variables

The Andersen model of healthcare utilization is widely used in health care utilization and health outcomes, and the model includes predisposing factors, enabling factors, need-based factors, health behaviors, and health outcomes of older adults [38, 39]. In our study, we focused on health behaviors (HCBS use) and health outcomes (loneliness). To identify association between the use of HCBS and loneliness, Andersen’s conceptual framework, in conjunction with the existing literature, guides the selection of confounding factors in the analysis. This includes considering predisposing factors, enabling factors, and needs-based factors. Predisposing factors refer to demographic characteristics that might influence the experience of loneliness, such as age, gender, education, and marital status. Enabling factors refer to resources that might impact feelings of loneliness, including area of residence, cash assistance, socioeconomic status, receipt of family care, and social activity. Need-based factors refer to individuals’ perceived and objective healthcare needs, which might contribute to heightened feelings of loneliness. These include self-rated health status, chronic pain, ADL disability, IADL disability, and outpatient/inpatient care received in the past year. Appendix Table 1 shows the assignment of the variables.

We considered sample selection bias in HCBS use. Selection bias occurs when the selection of participants into a study is not random, leading to systematic differences between the treatment (older adults who utilized HCBS) and control groups (older adults who did not utilize HCBS). As an example, having a higher socioeconomic status or a history of stroke can increase the likelihood of older individuals utilizing HCBS, leading to biased estimates of the association between HCBS and loneliness. In observational studies, propensity scores were used to adjust for confounding [40, 41]. Rubin recommends including as many covariates as possible in models predicting propensity to seek care, even those that are weak predictors of receiving care, to minimize the possibility of selection bias [42]. Therefore, on the basis of the above propensity factors, enabling factors, and need factors, we empirically additionally added the variables: diagnoses of diabetes, cancer, chronic lung disease, liver disease, heart disease, stroke, kidney disease, digestive disease, psychiatric disease, memory-related disease, arthritis, and asthma, in the analyses of propensity score matching (PSM).

### Statistical analysis

First, descriptive statistics were used to describe sociodemographic characteristics, HCBS use, and loneliness (Table 1). Then, we performed chi-squared tests to compare characteristics between participants who did and did not report loneliness (Table 2).

**Table 1:** Descriptive statistics of the characteristics of older people with functional limitations (n = 2188)

Variable	N (%)		Variable	N (%)	
**HCBS use**	No	1,704(77.9)	**Loneliness**	No	1,187(54.2)
	Yes	484(22.1)		Yes	1,001(45.8)
**Age**	65–69 years old	834(38.1)	**Gender**	Female	1,320(60.3)
	70–74 years old	590(27.0)		Male	868(39.7)
	≥ 75 years old	764(34.9)			
**Education**	Lower than primary school	1,429(65.3)	**Marital status**	Couple	1,537(70.2)
	Primary school or above	759(34.7)		Single	651(29.8)
**Area of residence**	Rural	1,810(82.7)	**Socioeconomic status**	Quintile1(lowest)	565(25.8)
	Urban	378(17.3)		Quintile2	603(27.6)
**Cash assistance**	No	1,846(84.4)		Quintile3	477(21.8)
	Yes	342(15.6)		Quintile4 (highest)	543(24.8)
**Receipt of family care**	No	918(42.0)	**Social activity**	No	1,264(57.8)
	Yes	1,270(58.0)		Yes	924(42.2)
**Self-rated health status**	Health	201(9.2)	**Chronic pain**	No	421(19.2)
	Unhealth	1,987(90.8)		Yes	1,767(80.8)
**ADL disability**	No	664(30.4)	**IADL disability**	No	484(22.1)
	1–2 items	1,072(49.0)		1–2 items	1,117(51.1)
	≥ 3 items	452(20.6)		≥ 3 items	587(26.8)
**Outpatient care received in the past year**	No	1,737(79.4)	**Inpatient care received in the past year**	No	1,533(70.1)
	Yes	451(20.6)		Yes	655(29.9)

**Table 2:** Baseline sample characteristics by loneliness of older people with functional limitations (n = 2188)

Characteristics	Loneliness		*χ*2	*P* value
	No	Yes		
**HCBS use**				
No	905(76.2)	799(79.8)	4.03	0.045
Yes	282(23.8)	202(20.2)		
**Predisposing factors**				
**Age**				
65–69	448(37.7)	386(38.6)	2.87	0.238
70–74	337(28.4)	253(25.2)		
≥ 75	402(33.9)	362(36.2)		
**Gender**				
Female	661(55.7)	659(65.8)	23.36	< 0.001
Male	526(44.3)	342(34.2)		
**Education**				
Lower than primary school	745(62.8)	684(68.3)	7.43	0.006
Primary school or above	442(37.2)	317(31.7)		
**Marital status**				
Couple	932(78.5)	605(60.4)	84.91	< 0.001
Single	255(21.5)	396(39.6)		
**Enabling factors**				
**Area of residence**				
Rural	959(80.8)	851(85.0)	6.77	0.009
Urban	228(19.2)	150(15.0)		
**Cash assistance**				
No	1,024(86.3)	822(82.1)	7.09	0.008
Yes	163(13.7)	179(17.9)		
**Socioeconomic status**				
Q1 (The poorest)	296(24.9)	269(26.9)	1.21	0.749
Q2	331(27.9)	272(27.1)		
Q2	265(22.3)	212(21.2)		
Q3 (The richest)	295(24.9)	248(24.8)		
**Receipt of family care**				
No	484(40.8)	434(43.4)	1.48	0.223
Yes	703(59.2)	567(56.6)		
**Social activity**				
No	677(57.0)	587(58.6)	0.57	0.448
Yes	510(43.0)	414(41.4)		
**Need-based factors**				
**Self-rated health status**				
Health	123(10.4)	78(7.8)	4.29	0.038
Unhealth	1,064(89.6)	923(92.2)		
**Chronic pain**				
No	287(24.2)	134(13.4)	40.70	< 0.001
Yes	900(75.8)	867(86.6)		
**ADL disability**				
No	402(33.9)	262(26.2)	38.44	< 0.001
1–2 items	595(50.1)	477(47.6)		
≥ 3 items	190(16.0)	262(26.2)		
**IADL disability**				
No	292(24.6)	192(19.2)	32.69	< 0.001
1–2 items	634(53.4)	483(48.2)		
≥ 3 items	261(22.0)	326(32.6)		
**Outpatient care received in the past years**				
No	942(79.4)	795(79.4)	0.001	0.972
Yes	245(20.6)	206(20.6)		
**Inpatient care received in the past year**				
No	852(71.8)	681(68.0)	3.63	0.057
Yes	335(28.2)	320(32.0)		

Second, binary logistic regression was performed to explore associations between HCBS use and loneliness. We evaluated several progressive models. Model 1 was unadjusted; Model 2 included predisposing factors and HCBS use; Model 3 included predisposing factors, enabling factors, and HCBS use; and the final model added need factors (Model 4) (Table 3). To check for multicollinearity, we calculated the variance inflation factor (VIF).

**Table 3:** Association between HCBS use and Loneliness in China: binary logistic regression

	Model 1 (n = 2188)	Model 2 (n = 2188)	Model 3 (n = 2188)	Model 4 (n = 2188)
OR	0.81**	0.78**	0.80**	0.81**
95%CI	(0.66–0.99)	(0.63–0.96)	(0.64–0.98)	(0.63–0.99)

**Notes**: ∗∗∗ *p* < 0.01, ∗∗ *p* < 0.05, ∗ *p* < 0.1, respectively indicate that the estimated results are significant at 1%, 5%, and 10% levels. Model 1: No confounding adjustment; Model 2 was adjusted for age, gender, education, marital status; Model 3 was adjusted for age, gender, education, marital status, area of residence, cash assistance, socioeconomic status, receipt of family care, social activity; Model 4 was adjusted for age, gender, education, marital status, area of residence, cash assistance, socioeconomic status, receipt of family care, social activity, self-rated health status, chronic pain, ADL disability, IADL disability, outpatient care received in the past years, inpatient care received in the past years

Third, propensity score matching (PSM) was used to further examine the associations between HCBS use and loneliness. The core idea of propensity score matching (PSM) is counterfactual inference by sampling from a large reservoir of potential controls to create a control group of modest size in which the data sets have similar distributions of confounders in the treated and non-treated groups [40]. The two groups, the receiving HCBS group and the non-receiving HCBS group, are as homogeneous and comparable as possible with respect to the remaining condition factors, except for the treatment factors, thus achieving the goal of minimizing selection and confounding bias.

PSM is a three-step process. In the first stage, a logistic regression model was employed to calculate propensity scores (PS) to adjust for confounding. In the second step, the propensity scores derived in the first step were used to match subjects with similar propensity scores to the treatment group sample from the large sample of potential controls for paired analysis. In this stage, quality assessment of matching was necessary to analyze the covariate balance between older adults who used HCBS and those who did not. The matching results were considered successful if there were no significant differences in the covariates between the groups of older adults who used HCBS and those who did not, after matching. In the third step, which built on the second step of matched samples, the Average Treatment Effect on the Treatment (ATT) was calculated to examine whether the use of HCBS was associated with a reduction in loneliness (Table 4). We selected three algorithms: the 1:1 nearest neighbor (without replacement) matching algorithm, the kernel matching algorithm, and the caliper matching algorithm for PSM, to ensure that the estimated results were more robust.

**Table 4:** Estimated ATT of HCBS use on the loneliness of older people with functional limitations

Treatment Variables	Matching algorithm	Treated	Control	*ATT*	Standard Errors	*T*-value
**HCBS use**	Nearest neighbor matching	0.417	0.500	-0.083	0.035	-2.37**
	Kernel matching	0.417	0.467	-0.049	0.025	-1.94*
	Caliper matching	0.417	0.422	-0.046	0.026	-1.78*

**Notes:** ∗∗∗ *p* < 0.01, ∗∗ *p* < 0.05, ∗ *p* < 0.1, respectively indicate that the estimated results are significant at 1%, 5%, and 10% levels

All analyses were conducted using Stata 15.0 software. In our study, we excluded individuals who had missing values for any of the main variables. The significance level was set at *P* value less than 0.1; all *P* values were two-sided. All results from logistic regression were reported as odds ratios (ORs) and 95% confidence intervals (95% CIs). This study followed the Strengthening the Reporting of Observational Studies in Epidemiology (STROBE) reporting guidelines [43].

## Results

### Descriptive analysis

A total of 2,188 older adults with functional limitations participated in this study; 38.1% were aged 65–69 years old, 27.0% were aged 70–74 years old, and 35.0% were aged ≥ 75 years old, approximately. Most were female (60.3%), 70.3% were married, and 82.7% were living in a rural area. The majority of participants (90.8%) reported that they were unhealthy. Only 22.1% of participants reported having used HCBS. Feeling lonely was reported by 1,001 (45.8%) participants. More information is provided in Table 1.

Table 2 presents significant differences in receipt of HCBS, gender, education, marital status, living area, cash assistance, self-rated health status, chronic pain, ADL disability, IADL disability, diabetes, chronic lung disease, and memory related disease between those who felt lonely and those who did not feel lonely. A majority of the participants who felt lonely were not receiving HCBS (79.8%), were female (65.8%), were living in a rural area (85.0%), and had finished lower than primary school (68.3%).

### Association between HCBS use and loneliness before propensity score matching

Table 3 shows that participants who received HCBS were less likely to experience loneliness than their peers who did not receive HBCS (OR = 0.81, 95% CI = 0.63–0.99, *p* = 0.048) after adjustment for confounders. The VIFs of the independent variables in model 4 were all less than 1.4, indicating minimal multicollinearity among the independent variables (Appendix Table 2).

### Association between HCBS use and loneliness after propensity score matching estimates

Only a small number of samples were lost in the matching process, and most samples in the recipient group found matching samples with similar PS in the potential control group (Appendix Fig. 2). The confounding variables were well balanced between the groups after matching, with no significant differences (Appendix Fig. 1, Appendix Table 3, Appendix Fig. 3).

We estimated the *ATT* of receiving HCBS on the loneliness of older people with functional limitations using three matching algorithms, and the results are presented in Table 4. The result of the nearest neighbor matching algorithm showed that receiving HCBS was significantly associated with decreased likelihood of reporting loneliness in the participants, at the significance level of 5% (*T* = -2.37). The results of the kernel matching method and the caliper matching algorithm showed a decrease in the likelihood of reporting loneliness in the older adults with functional limitations who used HCBS compared with those who did not, at the significance level of 10% (*T* = -1.94; *T* = -1.78, respectively). This suggests robust evidence supporting that among people with functional limitations, HCBS use was associated with less loneliness.

## Discussion

As China’s population ages, the blueprint for China’s emerging long-term care system consists of three tiers of care services: family care as the “foundation,” HCBS as the “backing,” and institutional care as the “support” to meet the growing needs of older adults, including not only physiological but also mental health needs [44]. To our knowledge, this is the first study to use nationally representative data to provide research evidence on loneliness in Chinese older adults with functional limitations and to investigate the association between HCBS use and loneliness among this population in the home setting.

In our study, 46% of Chinese older adults with functional limitations reported feeling lonely, which is higher than the prevalence of loneliness among both older adults aged 65 years or older in Europe (20-34%) [45] and older adults aged 70 years in the United States (25-29%) [46], and much higher than the prevalence of loneliness among Chinese older adults (65 years and older) in the general population (4-24%) [47]. The possible reasons for this are that older adults with functional limitations are more likely to have risk factors, such as low quality of social relationships, poor mental health, cognitive deficits, and communication difficulties, that may lead to or exacerbate loneliness [48]. This suggests that we need to recognize loneliness as a major health problem for older adults with functional limitations and emphasize the need for a deeper understanding of the nature of loneliness in this population.

Regarding the use of HCBS, our study found that less than a quarter of Chinese older adults with functional limitations reported using HCBS in 2018. This is lower than the overall utilization rate of HCBS in China (34%) reported in previous studies [49], and lower than the utilization of HCBS in the United States (37%) [50]. In recent years, American policy and payment reforms, such as the increasing availability of insurance coverage for HCBS, have led many states to expand options for HCBS. Previous surveys indicate that more than 9 million U.S. residents rely on HCBS for assistance with daily tasks [50]. In China, although the use of HCBS is increasing among the general population, it has been established that HCBS are not widely available [49].

The following reasons may exist for the low utilization rate of HCBS in China. From the perspective of care providers, the possible reasons include, first, that the workforce of HCBS has not kept pace with the growing need for HCBS [51] and second, that there has been reduced financial support from the government. Also, to date, policy initiatives to support HCBS have been largely confined to urban areas, and even there, the number of beneficiaries remains relatively small [20]. From the perspective of the care recipient, access to HCBS is less accessible for older adults with disabilities. To qualify for HCBS, people with functional limitations must meet strict health conditions or age requirements, rather than being considered based on their needs.

Our results showed that compared with participants who did not receive HCBS, people who received HCBS had a lower risk of feeling lonely. As we know, older adults with functional limitations are at great risk of feeling lonely due to a decline in social participation, loss of autonomy, and potentially decreasing social relationships [52]. Possible mechanisms underlying the relationship between HCBS use and loneliness include how HCBS may help expand the social network of older adults with disabilities and increase their opportunities for social participation [23, 24]. In terms of the subjective experience of individuals, HCBS help older adults with disabilities perceive subjective increased availability of support [53] and make meaningful social connections with professionals, while receiving the emotional support of close family and friends [54, 55].

Given the global challenge in identifying accurate and effective interventions to prevent and alleviate loneliness [56], our findings hold particular relevance for policymakers and HCBS providers. Investigations into the lack of effectiveness of current interventions aimed at alleviating loneliness have revealed that the most promising strategies are those that adapt to the local environment and encompass a holistic community context [57, 58]. Therefore, policymakers and HCBS providers should increase the availability of HCBS delivered to support population-based efforts, simultaneously addressing the issue of loneliness among homebound older adults with functional limitations.

Our study has several limitations. First, we used self-reported measures of HCBS use, loneliness, and some covariates (e.g., social activity, chronic pain), which may lead to misreporting. Nevertheless, a self-reported, single-item question about loneliness has been shown to be highly correlated with a widely used measure of loneliness, the UCLA Loneliness Scale [59]. Second, the cross-sectional nature of our data set does not allow us to make causal inferences. Third, our study considers only HCBS use as an intervention and does not address a range of conditions such as the quality of care content, the duration of care, or the structure and capacity of the service provider. Thus, future evaluations need to focus on identifying the most effective model of care, considering factors such as combinations of care, intensity and duration of care, group versus individual approaches, and building high-quality evidence across different administrative settings. Fourth, it is difficult to study the long-term dynamic influence of HCBS use on loneliness among older people with functional limitations using cross-sectional data. Individuals with severe cognitive problems were less likely to personally participate in the survey, which may limit the extrapolation of our findings to older adults with severe cognitive impairment.

Furthermore, some relevant variables may not be included in this study that could impact the association between home and community-based services (HCBS) and loneliness. This limitation means that the outcomes might be influenced by factors we did not account for. It is also important to note that our study focuses specifically on ADL/IADL functional limitations and does not include cognitive disability screening for the participants. Additionally, Tibet, Taiwan, Hong Kong, and Macao were not included in the survey. Given these factors, care must be taken to consider these potential biases when interpreting our study’s results.

## Conclusions

Loneliness was common among older people with functional limitations in China, and the proportion of HCBS recipients was low. Older people with functional limitations who received HCBS were associated with feeling less lonely. Public health strategies are needed to further expand and improve access to HCBS to promote and reduce loneliness among older persons with functional limitations.

### Electronic supplementary material

Below is the link to the electronic supplementary material.

Supplementary Material 1: Appendix Table 1. Codes of the characteristics of older people with functional limitations. Appendix Table 2. Variance inflation factor (VIF). Appendix Figure 1. Nuclear density distribution of propensity scores of the recipient group and non-recipient group before and after matching. Appendix Figure 2. Histogram of common value ranges of propensity scores. Appendix Table 3. Balance test results. Appendix Figure 3. Normalized deviation graph before and after matching

## Data Availability

The data that support the findings of this study are available from the Institute of Social Science Survey at the Peking University, and can be found on their website: https://charls.pku.edu.cn/.

## References

[1] The Lancet (2022). Population ageing in China: crisis or opportunity?. Lancet.

[2] Wang J, Wang Q, Hou XY (2021). Spousal concordance in the Development of Functional Limitations among married adults in China. JAMA Netw Open.

[3] The Chinese State Council. There are about 33 million disabled elderly people in urban and rural areas, accounting for 19% of the total elderly population | China. 2011 [Available from: http://www.gov.cn/jrzg/2011-03/02/content_1814376.htm 2011. Accessed on 3 Aug 2023].

[4] The Chinese State Council. Improve the elderly health support system | China. 2022 [Available from: http://www.gov.cn/xinwen/2022-02/22/content_5675059.htm 2022. Accessed on 3 Age 2023].

[5] Hu B (2019). Projecting future demand for informal care among older people in China: the road towards a sustainable long-term care system. Health Econ Policy Law.

[6] Mithen J, Aitken Z, Ziersch A (2015). Inequalities in social capital and health between people with and without disabilities. Soc Sci Med.

[7] Luhmann M, Hawkley LC (2016). Age differences in loneliness from late adolescence to oldest old age. Dev Psychol.

[8] Emerson E, Fortune N, Llewellyn G (2021). Loneliness, social support, social isolation and wellbeing among working age adults with and without disability: cross-sectional study. Disabil Health J.

[9] Surkalim DL, Luo M, Eres R (2022). The prevalence of loneliness across 113 countries: systematic review and meta-analysis. BMJ.

[10] Guo L, An L, Luo F (2021). Social isolation, loneliness and functional disability in Chinese older women and men: a longitudinal study. Age Ageing.

[11] Shankar A, McMunn A, Banks J (2011). Loneliness, social isolation, and behavioral and biological health indicators in older adults. Health Psychol.

[12] Harrington KD, Vasan S, Kang JE (2023). Loneliness and cognitive function in older adults without dementia: a systematic review and Meta-analysis. J Alzheimers Dis.

[13] Gale CR, Westbury L, Cooper C (2018). Social isolation and loneliness as risk factors for the progression of frailty: the English Longitudinal Study of Ageing. Age Ageing.

[14] Lara E, Moreno-Agostino D, Martín-María N (2020). Exploring the effect of loneliness on all-cause mortality: are there differences between older adults and younger and middle-aged adults?. Soc Sci Med.

[15] Newall N, McArthur J, Menec VH (2015). A longitudinal examination of social participation, loneliness, and use of physician and hospital services. J Aging Health.

[16] Gerst-Emerson K, Jayawardhana J (2015). Loneliness as a public health issue: the impact of loneliness on health care utilization among older adults. Am J Public Health.

[17] Feng ZL, Glinskaya E, Chen HT (2020). Long-term care system for older adults in China: policy landscape, challenges, and future prospects. Lancet.

[18] Xu Q, Chow JC (2011). Exploring the community-based service delivery model: Elderly care in China. Int Social Work.

[19] Ruiz S, Snyder LP, Rotondo C (2017). Innovative home visit Models Associated with Reductions in costs, hospitalizations, and Emergency Department Use. Health Aff (Millwood).

[20] Feng Z, Liu C, Guan X (2012). China’s rapidly aging population creates policy challenges in shaping a viable long-term care system. Health Aff (Millwood).

[21] General Office of the Ministry of Civil Affairs General Office of the Ministry of Finance Notice on the Organization and Implementation of the 2022 Home and Community-based Basic Senior Care Service Enhancement Project | China. 2022 [Available from: http://www.gov.cn/zhengce/zhengceku/2022-09/25/content_5711797.htm. Accessed 11 Feb 2023].

[22] Opinions on the comprehensive promotion of home care services | China. 2008 [Available from: http://www.gov.cn/zwgk/2008-02/25/content_899738.htm. Accessed 11 Feb 2023].

[23] Wang Q, Fan K, Li P (2022). Effect of the Use of Home and Community Care Services on the Multidimensional Health of older adults. Int J Environ Res Public Health.

[24] Su Q, Wang H, Fan L (2023). The impact of home and community care services pilot program on healthy aging: a difference-in-difference with propensity score matching analysis from China. Arch Gerontol Geriatr.

[25] Brumley R, Enguidanos S, Jamison P (2007). Increased satisfaction with care and lower costs: results of a randomized trial of in-home palliative care. J Am Geriatr Soc.

[26] Lykke S, Handberg C (2019). Experienced loneliness in Home-Based Rehabilitation: perspectives of older adults with disabilities and their Health Care Professionals. Glob Qual Nurs Res.

[27] Honigh-de Vlaming R, Haveman-Nies A, Heinrich J (2013). Effect evaluation of a two-year complex intervention to reduce loneliness in non-institutionalised elderly dutch people. BMC Public Health.

[28] Cattan M, White M, Bond J (2005). Preventing social isolation and loneliness among older people: a systematic review of health promotion interventions. Ageing Soc.

[29] Findlay RA (2003). Interventions to reduce social isolation amongst older people: where is the evidence?. Ageing Soc.

[30] China Health and Retirement Longitudinal Study, About CHARLS. 2018. | China. 2018 [Available from: http://charls.pku.edu.cn/pages/about/111/zh-cn.html. Accessed: 5 Feb 2023].

[31] Katz S, Ford AB, Moskowitz RW (1963). The index of ADL:a standardized measure of Biological and psychological function. JAMA.

[32] Lawton MP, Brody EM (1969). Assessment of older people: self-maintaining and instrumental activities of daily living. Gerontologist.

[33] Xu T, Huang Z, Huang Y (2023). Association between home and community-based services and depressive symptoms in chinese older adults: a multilevel analysis. BMC Public Health.

[34] Jiang H, Liu Z (2023). Community home elderly care services, multidimensional health and social participation of chronically ill elderly-empirical analysis based on propensity score matching and multiple mediation analysis. Front Public Health.

[35] Zhao M, Gao J, Li M (2019). Relationship between loneliness and Frailty among older adults in nursing Homes: the Mediating Role of Activity Engagement. J Am Med Dir Assoc.

[36] Luo Y, Waite LJ (2014). Loneliness and mortality among older adults in China. J Gerontol B Psychol Sci Soc Sci.

[37] Luanaigh CO, Lawlor BA (2008). Loneliness and the health of older people. Int J Geriatr Psychiatry.

[38] Andersen R, Newman JF (1973). Societal and individual determinants of medical care utilization in the United States. Milbank Q.

[39] Andersen RM (1995). Revisiting the behavioral model and access to medical care: does it matter?. J Health Soc Behav.

[40] Rosenbaum PR, Rubin DB (1983). The central role of the propensity score in observational studies for causal effects. Biometrika.

[41] Rosenbaum PR, Rubin DB (1985). Constructing a control-group using multivariate matched sampling methods that incorporate the propensity score. Am Stat.

[42] Rubin DB (1997). Estimating causal effects from large data sets using propensity scores. Ann Intern Med.

[43] Knottnerus A, Tugwell P (2008). STROBE—A checklist to STrengthen the reporting of OBservational studies in Epidemiology. J Clin Epidemiol.

[44] Feng D, Ji L, Xu L (2014). Mediating effect of social support on the association between functional disability and psychological distress in older adults in rural China: does age make a difference. PLoS ONE.

[45] Yang K, Victor C (2011). Age and loneliness in 25 european nations. Ageing Soc.

[46] Ong AD, Uchino BN, Wethington E (2016). Loneliness and health in older adults: a Mini-Review and Synthesis. Gerontology.

[47] Gao Q, Prina AM, Prince M (2021). Loneliness among older adults in Latin America, China, and India: prevalence, Correlates and Association with Mortality. Int J Public Health.

[48] Tomida K, Lee S, Makino K et al. Association of Loneliness with the incidence of disability in older adults with hearing impairment in Japan. JAMA Otolaryngol Head Neck Surg. 2023;e230309. 10.1001/jamaoto.2023.0309 PMC10080402 37022721

[49] Li Z, Xuan M, Gao Y (2023). Trends in the availability of community-based home visiting services for oldest-old in China, 2005–2018. BMJ Open.

[50] Van Houtven CH, Konetzka RT, Taggert E (2020). Informal and formal Home Care for older adults with disabilities increased, 2004-16. Health Aff (Millwood).

[51] Kreider AR, Werner RM (2023). The Home Care Workforce has not kept Pace with Growth in Home and Community-Based Services. Health Aff (Millwood).

[52] Hawkley LC, Cacioppo JT (2010). Loneliness matters: a theoretical and empirical review of consequences and mechanisms. Ann Behav Med.

[53] Kerr CW, Tangeman JC, Rudra CB (2014). Clinical impact of a home-based palliative care program: a hospice-private payer partnership. J Pain Symptom Manage.

[54] Masi CM, Chen HY, Hawkley LC (2011). A meta-analysis of interventions to reduce loneliness. Pers Soc Psychol Rev.

[55] Yanguas J, Pinazo-Henandis S, Tarazona-Santabalbina FJ (2018). The complexity of loneliness. Acta Biomed.

[56] Akhter-Khan SC, Au R (2020). Why loneliness interventions are unsuccessful: a call for Precision Health. Adv Geriatr Med Res.

[57] Gardiner C, Geldenhuys G, Gott M (2018). Interventions to reduce social isolation and loneliness among older people: an integrative review. Heal Soc Care Community.

[58] Poscia A, Stojanovic J, La Milia DI (2018). Interventions targeting loneliness and social isolation among the older people: an update systematic review. Exp Gerontol.

[59] Russell D, Peplau LA, Ferguson ML (1978). Developing a measure of loneliness. J Pers Assess.

