# Supplementary material for: Use of home and community-based services and loneliness in older people with functional limitations: a cross-sectional study
Source: BMC Psychiatry. 2023 Oct 4;23:717. doi: 10.1186/s12888-023-05225-6 (PMC10548717; doi:10.1186/s12888-023-05225-6)
Supplement: Supplementary file 1 — Supplementary Material 1: Appendix Table 1. Codes of the characteristics of older people with functional limitations. Appendix Table 2. Variance inflation factor (VIF). Appendix Figure 1. Nuclear density distribution of propensity scores of the recipient group and non-recipient group before and after matching. Appendix Figure 2. Histogram of common value ranges of propensity scores. Appendix Table 3. Balance test results. Appendix Figure 3. Normalized deviation graph before and after matching [file 12888_2023_5225_MOESM1_ESM.docx]

# Appendix – Supplementary Material

***Appendix Table 1.* Codes of the characteristics of older people with functional limitations**

| **Variable** | **Codes/definition** | |
| --- | --- | --- |
| **Receipt of home and community-based services** | Binary variable | 0=No |
|  |  | 1=Yes |
| **Loneliness** | Binary variable | 0=No |
|  |  | 1=Yes |
| **Predisposing factors** | | |
| **Age** | Categorical variable | 0=65-69 years old; |
|  |  | 1=70-74 years old; |
|  |  | 2= ≥75 years old |
| **Gender** | Binary variable | 0=Female |
|  |  | 1=Male |
| **Education** | Binary variable | 0=Lower than primary school |
|  |  | 1=Primary school or above |
| **Marital status** | Binary variable | 0=Couple |
|  |  | 1=Single |
| **Enabling factors** | | |
| **Area of residence** | Binary variable | 0=Rural |
|  |  | 1=Urban |
| **Cash assistance** | Binary variable | 0=No |
|  |  | 1=Yes |
| **Socioeconomic status** | Categorical variable | 0=Quintile1(lowest) |
|  |  | 1= Quintile2 |
|  |  | 2= Quintile3 |
|  |  | 3= Quintile4 (highest) |
| **Receipt of family care** | Binary variable | 0=No |
|  |  | 1=Yes |
| **Social activity** | Binary variable | 0=No |
|  |  | 1=Yes |
| **Need-based factors** | | |
| **Self-rated health status** | Binary variable | 0=Health |
|  |  | 1=Unhealth |
| **Chronic pain** | Binary variable | 0=No |
|  |  | 1=Yes |
| **ADL disability** | Categorical variable | 0=No |
|  |  | 1=1-2 items |
|  |  | 2= ≥3 items |
| **IADL disability** | Categorical variable | 0=No |
|  |  | 1=1-2 items |
|  |  | 2= ≥3 items |
| **Outpatient care received in the past year** | Binary variable | 0=No |
|  |  | 1=Yes |
| **Inpatient care received in the past year** | Binary variable | 0=No |
|  |  | 1=Yes |
| **Hypertension** | Binary variable | 0=No |
|  |  | 1=Yes |
| **Dyslipidemia** | Binary variable | 0=No |
|  |  | 1=Yes |
| **Diabetes** | Binary variable | 0=No |
|  |  | 1=Yes |
| **Cancer** | Binary variable | 0=No |
|  |  | 1=Yes |
| **Chronic lung disease** | Binary variable | 0=No |
|  |  | 1=Yes |
| **Liver disease** | Binary variable | 0=No |
|  |  | 1=Yes |
| **Heart disease** | Binary variable | 0=No |
|  |  | 1=Yes |
| **Stroke** | Binary variable | 0=No |
|  |  | 1=Yes |
| **Kidney disease** | Binary variable | 0=No |
|  |  | 1=Yes |
| **Digestive disease** | Binary variable | 0=No |
|  |  | 1=Yes |
| **Psychiatric disease** | Binary variable | 0=No |
|  |  | 1=Yes |
| **Memory related disease** | Binary variable | 0=No |
|  |  | 1=Yes |
| **Arthritis** | Binary variable | 0=No |
|  |  | 1=Yes |
| **Asthma** | Binary variable | 0=No |
|  |  | 1=Yes |

**Notes**: Disability was characterized as individuals reporting difficulty with one or more activities of daily living (ADL or IADL), regardless of whether assistance was needed (Katz SJ, 2000; Tas U, 2007; Qiao Y, 2021). Difficulties in performing activities are recognized as a form of disability, as supported by previous research (Katz SJ, 2000; Tas U, 2007; Qiao Y, 2021). In our study, we used the measures of disability, specifically ADL and IADL difficulties, as confounding factors. This decision was informed by the understanding that experiencing ADL and IADL difficulties may lead to reduced engagement in social activities, which has been associated with feelings of loneliness (Philip KEJ, 2020; Kanamori S, 2014).

***Appendix Table 2. Variance inflation factor (VIF)***

| **Variable** | **VIF** |  |
| --- | --- | --- |
| **IADL disability** | 1.37 |  |
| **Receipt of family care** | 1.34 |  |
| **Marital status** | 1.22 |  |
| **Gender** | 1.20 |  |
| **Education** | 1.20 |  |
| **Age** | 1.19 |  |
| **Area of residence** | 1.17 |  |
| **Socioeconomic status** | | 1.13 |
| **Chronic pain** | 1.11 |  |
| **Inpatient care received in the past year** | 1.10 |  |
| **Self-rated health status** | 1.07 |  |
| **ADL disability** | 1.07 |  |
| **Social activity** | 1.06 |  |
| **Outpatient care received in the past year** | 1.05 |  |
| **Cash assistance** | 1.03 |  |
| **Receipt of HCBS** | 1.02 |  |


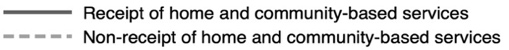


**X: propensity score**

***Appendix*** ***Figure 1.*** ***Nuclear density distribution of*** ***propensity scores of the recipient group and non-recipient*** ***group before and after matching***

**Appendix Figure 1** shows the kernel density function distribution of the propensity score before and after matching between the recipient and non-recipient groups, indicating that after matching there was a large range of overlap in the common support domains of both the recipient and non-recipient groups.

***Appendix Figure 2. Histogram of common value ranges of propensity scores***

**Appendix Figure 2** indicated that only a small number of samples were lost in the matching process, and most samples in the recipient group found matching samples with similar PS in the potential control group (non-recipient group).

***Appendix Table 3.*** ***Balance test results***

| **Variable** | **Matching status** | **Mean** | | **Bias (%)** | **%reduct \|bias\|** | **T-test** | ***p*>\|t\|** |
| --- | --- | --- | --- | --- | --- | --- | --- |
|  |  | **Treated** | **Control** |  |  |  |  |
| **Age** | Unmatched | 1.00 | 0.95 | 4.8 |  | 0.93 | 0.351 |
|  | Matched | 1.00 | 0.99 | 0.9 | 80.7 | 0.14 | 0.886 |
| **Gender** | Unmatched | 0.40 | 0.39 | 1.1 |  | 0.21 | 0.834 |
|  | Matched | 0.40 | 0.40 | -0.3 | 75.5 | -0.04 | 0.967 |
| **Education** | Unmatched | 0.35 | 0.34 | 3.4 |  | 0.66 | 0.509 |
|  | Matched | 0.35 | 0.35 | 0.6 | 82.2 | 0.09 | 0.926 |
| **Marital status** | Unmatched | 0.32 | 0.29 | 6.3 |  | 1.24 | 0.216 |
|  | Matched | 0.31 | 0.31 | 0.8 | 87.4 | 0.12 | 0.903 |
| **Area of residence** | Unmatched | 0.18 | 0.16 | 5.1 |  | 1.01 | 0.315 |
|  | Matched | 0.18 | 0.18 | 0.5 | 90.6 | 0.07 | 0.941 |
| **Cash assistance** | Unmatched | 0.15 | 0.15 | -1.9 |  | -0.38 | 0.707 |
|  | Matched | 0.15 | 0.14 | 0.6 | 69.9 | 0.09 | 0.927 |
| **Socioeconomic status** | Unmatched | 2.52 | 2.43 | 8.4 |  | 1.62 | 0.106 |
|  | Matched | 2.51 | 2.50 | 1.0 | 87.9 | 0.16 | 0.876 |
| **Receipt of family care** | Unmatched | 0.57 | 0.58 | -1.6 |  | -0.31 | 0.760 |
|  | Matched | 0.57 | 0.57 | -0.3 | 79.6 | -0.05 | 0.960 |
| **Social activity** | Unmatched | 0.49 | 0.40 | 19.1 |  | 3.72 | 0.000*** |
|  | Matched | 0.49 | 0.48 | 1.0 | 94.6 | 0.16 | 0.874 |
| **Self-rated health status** | Unmatched | 0.88 | 0.91 | -9.3 |  | -1.88 | 0.060* |
|  | Matched | 0.88 | 0.88 | -0.9 | 90.1 | -0.14 | 0.892 |
| **Chronic pain** | Unmatched | 0.80 | 0.80 | 0.8 |  | 0.15 | 0.883 |
|  | Matched | 0.80 | 0.81 | -0.7 | 7.2 | -0.11 | 0.913 |
| **ADL disability** | Unmatched | 1.00 | 1.00 | -2.7 |  | -0.52 | 0.605 |
|  | Matched | 1.00 | 1.00 | -0.3 | 87.7 | -0.05 | 0.959 |
| **IADL disability** | Unmatched | 0.14 | 0.12 | -7.9 |  | -1.53 | 0.125 |
|  | Matched | 0.13 | 0.14 | -0.7 | 91.1 | -0.11 | 0.913 |
| **Outpatient care received in the past year** | Unmatched | 0.22 | 0.20 | 6.0 |  | 1.18 | 0.240 |
|  | Matched | 0.22 | 0.22 | -1.3 | 78.9 | -0.19 | 0.848 |
| **Inpatient care received in the past year** | Unmatched | 0.30 | 0.29 | 0.6 |  | 0.12 | 0.901 |
|  | Matched | 0.29 | 0.29 | 0.2 | 75.7 | 0.02 | 0.981 |
| **Hypertension** | Unmatched | 0.14 | 0.12 | 6.0 |  | 1.20 | 0.232 |
|  | Matched | 0.13 | 0.14 | -2.0 | 66.1 | -0.31 | 0.758 |
| **Dyslipidemia** | Unmatched | 0.12 | 0.12 | 0.8 |  | 0.15 | 0.883 |
|  | Matched | 0.12 | 0.12 | -1.6 | -113.2 | -0.25 | 0.804 |
| **Diabetes** | Unmatched | 0.07 | 0.07 | -2.4 |  | -0.46 | 0.646 |
|  | Matched | 0.07 | 0.07 | 0.0 | 99.9 | 0.00 | 1.000 |
| **Cancer** | Unmatched | 0.02 | 0.02 | 3.7 |  | 0.75 | 0.452 |
|  | Matched | 0.02 | 0.02 | -0.6 | 82.7 | -0.10 | 0.922 |
| **Chronic lung disease** | Unmatched | 0.08 | 0.06 | 7.0 |  | 1.40 | 0.161 |
|  | Matched | 0.08 | 0.08 | -3.1 | 55.1 | -0.47 | 0.641 |
| **Liver disease** | Unmatched | 0.04 | 0.04 | 0.1 |  | 0.02 | 0.981 |
|  | Matched | 0.04 | 0.03 | 1.0 | -753.2 | 0.16 | 0.871 |
| **Heart disease** | Unmatched | 0.09 | 0.11 | -7.0 |  | -1.33 | 0.183 |
|  | Matched | 0.09 | 0.09 | -0.8 | 88.4 | 0.13 | 0.895 |
| **Stroke** | Unmatched | 0.12 | 0.09 | 9.9 |  | 1.98 | 0.047* |
|  | Matched | 0.11 | 0.11 | -0.3 | 97.0 | -0.04 | 0.965 |
| **Kidney disease** | Unmatched | 0.07 | 0.06 | 2.4 |  | 0.46 | 0.643 |
|  | Matched | 0.07 | 0.07 | -0.7 | 71.9 | -0.10 | 0.920 |
| **Digestive disease** | Unmatched | 0.08 | 0.08 | 1.0 |  | 0.20 | 0.842 |
|  | Matched | 0.08 | 0.08 | -1.0 | 6.8 | -0.15 | 0.883 |
| **Psychiatric disease** | Unmatched | 0.01 | 0.02 | -10.3 |  | -1.80 | 0.072* |
|  | Matched | 0.01 | 0.01 | -0.2 | 98.5 | -0.03 | 0.976 |
| **Memory related disease** | Unmatched | 0.05 | 0.05 | 2.4 |  | 0.47 | 0.641 |
|  | Matched | 0.05 | 0.05 | 0.2 | 89.8 | 0.04 | 0.971 |
| **Arthritis** | Unmatched | 0.07 | 0.08 | -2.8 |  | -0.53 | 0.598 |
|  | Matched | 0.07 | 0.07 | -0.7 | 74.9 | -0.11 | 0.914 |
| **Asthma** | Unmatched | 0.04 | 0.04 | 1.1 |  | 0.22 | 0.828 |
|  | Matched | 0.04 | 0.04 | 0.8 | 30.9 | 0.12 | 0.904 |

*p<0.05, **p<0.01, ***p<0.001 respectively indicate that the estimated results are significant at 1%, 5%, and 10% levels. Values in the table are means. “Bias (%)”: the standardized difference in means. “% reduct |bias|”: the percentage reduction of the bias through kernel matching. t-test was applied for mean comparisons of each variable between treated and control groups.

**Appendix Table 3** shows that before matching, the standardized deviation rates of confounding variables are high, and some confounding variables have significant differences between the recipient and non-recipient groups. After matching, the absolute values of standardized deviations of all confounding variables were less than 10% and the *P* values were greater than 0.1, indicating that there was no significant difference between confounding variables in the recipient and non-recipient groups, and the matching between the recipient and non-recipient groups passed the balance test.

***Appendix Figure 3. Normalized deviation graph before and after matching***

**Appendix Figure 3** shows that after matching, the absolute values of the standardized deviations of all confounding variables were less than 10%, for a more visual representation of the change in the characteristic difference of the confounding variables before and after matching, indicating a good balance test effect.

***References***

*Katz SJ, Kabeto M, Langa KM. Gender disparities in the receipt of home care for elderly people with disability in the United States. JAMA. 2000 Dec 20;284(23):3022-7. doi: 10.1001/jama.284.23.3022. PMID: 11122589.*

*Tas U, Verhagen AP, Bierma-Zeinstra SM, Odding E, Koes BW. Prognostic factors of disability in older people: a systematic review. Br J Gen Pract. 2007 Apr;57(537):319-23. PMID: 17394736; PMCID: PMC2043327.*

*Qiao Y, Liu S, Li G, Lu Y, Wu Y, Shen Y, Ke C. Longitudinal Follow-Up Studies on the Bidirectional Association between ADL/IADL Disability and Multimorbidity: Results from Two National Sample Cohorts of Middle-Aged and Elderly Adults. Gerontology. 2021;67(5):563-571. doi: 10.1159/000513930. Epub 2021 Jun 28. PMID: 34182559.*

*Kanamori S, Kai Y, Aida J, Kondo K, Kawachi I, Hirai H, Shirai K, Ishikawa Y, Suzuki K; JAGES Group. Social participation and the prevention of functional disability in older Japanese: the JAGES cohort study. PLoS One. 2014 Jun 12;9(6):e99638. doi: 10.1371/journal.pone.0099638. PMID: 24923270; PMCID: PMC4055714.*

*Philip KEJ, Polkey MI, Hopkinson NS, Steptoe A, Fancourt D. Social isolation, loneliness and physical performance in older-adults: fixed effects analyses of a cohort study. Sci Rep. 2020 Aug 17;10(1):13908. doi: 10.1038/s41598-020-70483-3. PMID: 32807857; PMCID: PMC7431531.*
